# Cardanol: toxicogenetic assessment and its effects when combined with cyclophosphamide

**DOI:** 10.1590/1678-4685-GMB-2015-0170

**Published:** 2016

**Authors:** Beatriz Ursinos Catelan Schneider, Alisson Meza, Adilson Beatriz, João Renato Pesarini, Pamela Castilho de Carvalho, Mariana de Oliveira Mauro, Caroline Bilhar Karaziack, Andréa Luiza Cunha-Laura, Antônio Carlos Duenhas Monreal, Renata Matuo, Dênis Pires de Lima, Rodrigo Juliano Oliveira

**Affiliations:** 1 Programa de Pós-Graduação em Saúde e Desenvolvimento na Região Centro-Oeste, Faculdade de Medicina "Dr. Hélio Mandetta", Universidade Federal de Mato Grosso do Sul, Campo Grande, MS, Brazil; 2 Centro de Estudos em Células-Tronco, Terapia Celular e Genética Toxicológica, Hospital Universitário "Maria Aparecida Pedrossian", Empresa Brasileira de Serviços Hospitalares, Campo Grande, MS, Brazil; 3 Programa de Pós-Graduação em Química, Instituto de Química, Universidade Federal de Mato Grosso do Sul, Campo Grande, MS, Brazil; 4 Programa de Mestrado em Farmácia, Centro de Ciências Biológicas e da Saúde, Universidade Federal de Mato Grosso do Sul, Campo Grande, MS, Brazil; 5 Programa de Doutorado em Biotecnologia e Biodiversidade - Rede Pró Centro-Oeste, Universidade Federal de Mato Grosso do Sul, Campo Grande, MS, Brazil

**Keywords:** phenolic lipid, antimutagenesis, micronucleus, comet assay, apoptosis

## Abstract

Cardanol is an effective antioxidant and is a compound with antimutagenic and antitumoral activity. Here, we evaluated the genotoxic and mutagenic potential of saturated side chain cardanol and its effects in combination with cyclophosphamide in preventing DNA damage, apoptosis, and immunomodulation. Swiss mice were treated with cardanol (2.5, 5 and 10 mg/kg) alone or in combination with cyclophosphamide (100 mg/kg). The results showed that cardanol is an effective chemopreventive compound, with damage reduction percentages that ranged from 18.9 to 31.76% in the comet assay and from 45 to 97% in the micronucleus assay. Moreover, cardanol has the ability to reduce the frequency of apoptosis induced by cyclophosphamide. The compound did not show immunomodulatory activity. A final interpretation of the data showed that, despite its chemoprotective capacity, cardanol has a tendency to induce DNA damage. Hence, caution is needed if this compound is used as a chemopreventive agent. Also, this compound is likely not suitable as an adjuvant in chemotherapy treatments that use cyclophosphamide.

## Introduction

Cancer is a chronic degenerative disease of high global prevalence. It is recognized as a key public health issue (Instituto Nacional do Câncer - INCA, 2014) and accounted for the death of 8.2 million people in 2012 according to the World Health Organization (WHO). These same institutions estimate that approximately 75 million people will have cancer in 2030, and 17 million deaths are likely to occur because of that disease worldwide (WHO, 2014).

Given this scenario, studies searching for natural compounds with the ability to protect DNA and aiming to clarify possible chemopreventive mechanisms are increasingly needed. Chemoprevention is defined as the systemic use of natural or synthetic chemical agents to reverse or suppress the transformation of premalignant lesions into malignant ones (Sporn, 1976). Such agents include substances with antioxidant (Miguel *et al.*, 2010), antigenotoxic (Skandrani *et al.*, 2010) and antimutagenic (Malini *et al.*, 2010) activity, and those able to activate DNA repair pathways (Duarte *et al.*, 2009). Also, there is an important need to find compounds without toxicity but with the ability to potentiate the antitumor effects of commercial chemotherapy and/or increase their selectivity (Navarro *et al.*, 2014; Carvalho *et al.*, 2015; Oliveira *et al.*, 2015). Thus, not only the chemoprotective properties are important to novel compounds but also the capability of these as adjuvants to chemotherapy.

**Figure 1:**
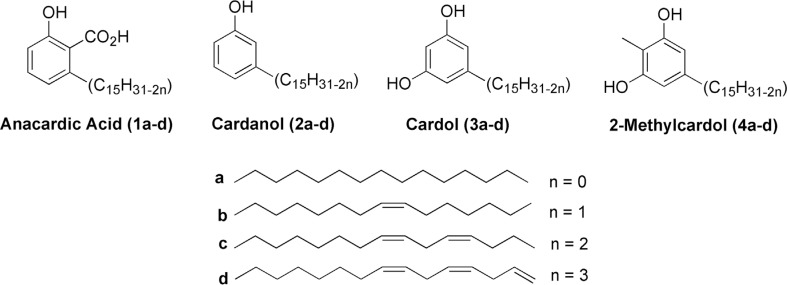
Structure of main components of Cashew nut shell liquid.

**Figure 2:**
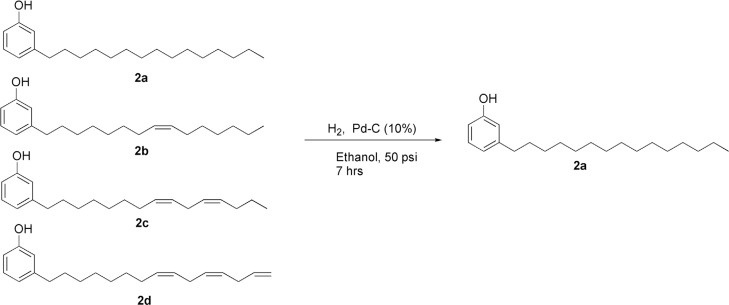
Catalytic hydrogenation of the cardanol mixture. (A) Saturated chain cardanol. (B-D) Unsaturated chain compounds.

In view of this, a strong candidate with protective and/or chemotherapeutic adjuvant potential is cashew nut shell liquid. Studies have shown that phenolic lipids derived from cashew nut shell liquid, such as anacardic acid, cardanol, cardol and 2-methylcardol (Figure 1) have antibacterial (Bouttier *et al.*, 2002; Kubo *et al.*, 2003, 2006), antioxidant and antimutagenic activities (Melo Cavalcante *et al.*, 2003; Rodrigues *et al.*, 2006; Trevisan *et al.*, 2006; De Lima *et al.*, 2008), and antitumor activities (Teerasripreecha *et al.*, 2012; Patel, 2016). Cardanol is a phenolic lipid with a long aliphatic chain joined to a phenolic ring (Figure 2) (Brady *et al.*, 2000; Baerson *et al.*, 2010; Stasiuk and Kozubek, 2010), which presents antibacterial (Begum *et al.*, 2002), larvicidal (Lomonaco *et al.*, 2009) and antitumor activities (Teerasripreecha *et al.*, 2012; Patel, 2016), and antioxidant properties (Trevisan *et al.*, 2006). This study aimed to evaluate, in a preclinical model, the genotoxic and mutagenic action of saturated side chain cardanol and its effects in combination with cyclophosphamide in preventing DNA damage, apoptosis and immunomodulation.

## Material and Methods

### Isolation of cardanol

Cardanol was obtained according to Srinivasa *et al.* (2002) with modifications. Five grams of technical cashew nut shell liquid (Cascaju Agroindustrial S/A; Lot 2001) was solubilized in 30 mL methanol and 20 mL ammonium hydroxide. The solution was mixed on a magnetic stirrer for 10 min. Cardanol was extracted with hexane (3 x 20 mL), and hexane phases were pooled and neutralized with 0.1 M HCl (2 x 20 mL), followed by evaporation of the solvent. Thin-layer chromatography was performed and the ultraviolet-scanned glass plates showed the presence of cardanols and other phenolic lipids. Liquid column chromatography on flash silica gel with the eluent consisting of 10:1 hexane:ethyl acetate (v/v) was performed to separate such compounds. Approximately 2.75 g (55% yields) unsaturated chain cardanols resulted from this process. The mixture of unsaturated chain cardanols was subjected to catalytic hydrogenation in a Parr 3911EG® hydrogenator (Parr Instruments, Moline IL, USA) to convert the unsaturated chain compounds (Figure 2B-D) into saturated chain cardanol (Figure 2A). A sample of 1.27 g cardanol was solubilized in 50 mL ethanol, together with 0.1 g palladium on activated carbon (Pd-C; 10%). The solution was hydrogenated under stirring for 7 h at 50 psi pressure and was subsequently filtered with celite. The product was prepurified by liquid column chromatography, with the same eluent previously used, and the product, 3-pentadecylphenol, was recrystallized in 90% ethanol. The white crystalline solid (MP: 51-52 °C, 1.08 g, 85% yields) was further analyzed by nuclear magnetic resonance (NMR) spectra (recorded in CDCl_3_ solution on a Bruker DPX300 spectrometer operating at 300 MHz for ^1^H). NMR spectra of ^1^H (see Supplementary Material Figure S1) and melting point were used as criteria of purity of the saturated cardanol.

### Experimental design

Forty sexually mature male *Swiss* mice *(Mus musculus)*, 8-10 weeks old, derived from the Central Animal Facility of the Center for Biological Sciences and Health, Federal University of Mato Grosso do Sul (Centro de Ciências Biológicas e Saúde da Universidade Federal de Mato Grosso do Sul, CCBS/UFMS) were used. The animals were split into eight experimental groups (n = 5). The mice were maintained in polypropylene boxes with wood shaving bedding and provided with commercial feed (Nutival®) and filtered water *ad libitum* throughout the experiment*.* Light and temperature were controlled using a 12 h photoperiod (12:12 h DL) with a temperature of 22 ± 2 °C and humidity of 55 ± 10% on a ventilated shelf (ALESCO®, Monte Mor, Brazil). The experiment was conducted according to the guidelines of the Universal Declaration of Animal Rights and with the approval of the Ethics Committee on Animal Use of UFMS (Protocol Number 399/2011).

Cardanol was diluted in 4% *Tween* 80 and subsequently in ethanol (1%). The compound was administered intraperitoneally (i.p.) at 2.5, 5.0 and 10.0 mg/kg body weight (b.w.),. The dose of 2.5 mg/kg was defined based on an experiment conducted by Wu *et al.* (2011) and subsequent higher doses were proposed by our research group. Cyclophosphamide (Fosfazeron®, Ítaca laboratory, REG. M.S. Number 1.26030056002-1; Batch 063020, Brazil) at a dose of 100 mg/kg b.w., administered i.p. in a single injection was used as a positive control (Navarro *et al.*, 2014).

For comparative purposes, all treatments including cardanol were performed using 4% Tween and 1% ethanol as vehicle. Conversely, those including cyclophosphamide used 0.9% saline as vehicle. The experimental groups and doses of the compounds are shown in Table 1. The treatment applications occurred simultaneously.

**Table 1:** Experimental groups and doses.

Treatment	Cardanol	Cyclophosphamide
Control	-	-
CP 100 mg/kg	-	+
Car 2.5 mg/kg	+	-
Car 5 mg/kg	+	-
Car 10 mg/kg	+	-
CP + Car 2.5 mg/kg	+	+
CP + Car 5 mg/kg	+	+
CP + Car 10 mg/kg	+	+

CP 100 mg/kg: Experimental group that received cyclophosphamide at the dose of 100 mg/kg;

Car 2.5 mg/kg: Experimental group that received cardanol at the dose of 2.5 mg/kg;

Car 5 mg/kg: Experimental group that received cardanol at the dose of 5 mg/kg;

Car 10 mg/kg: Experimental group that received cardanol at the dose of 10 mg/kg;

CP + Car 2.5 mg/kg: Experimental group that received cyclophosphamide (100 mg/kg) and cardanol at the dose of 2.5 mg/kg;

CP + Car 5 mg/kg: Experimental group that received cyclophosphamide (100 mg/kg) and cardanol at the dose of 5 mg/kg;

CP + Car 10 mg/kg: Experimental group that received cyclophosphamide (100 mg/kg) and cardanol at the dose of 10 mg/kg.

### Evaluation of biometric parameters

Animals were weighted before and 72 h after treatments. Weight gain was calculated by the difference between animal weight after and before treatments. Following 72 h of treatment, the animals were euthanized, and organs (kidneys, heart, liver, lungs and spleen) were collected and weighed. Relative organ weight was calculated as the ratio of each organ absolute weight to the animal's final weight.

### Comet assay

The alkaline comet assay was employed for genotoxicity evaluation. Twenty four hours after treatment, 20 μL of peripheral blood was collected for this assay. The analyzed cells were mainly leukocytes, and procedures of the comet assay were based on Singh *et al.* (1988). Analyses were performed by epifluorescence microscopy (Bioval®, Model L 2000A, São Paulo, Brazil) at 400x magnification with a 420-490 nm excitation and a 520 nm barrier filter. A total of 100 cells per animal were examined by visual analysis, and DNA fragment migration was determined according to comet class, as described by Kobayashi *et al.* (1995) with modifications (Oliveira *et al.*, 2015): class 0, intact nucleoid without tail; class 1, cell with tail less than the diameter of the nucleoid; class 2, tail size varying between one and two times the diameter of the nucleoid; class 3, tail size more than two times the diameter of the nucleoid. Apoptotic cells that showed a totally fragmented nucleus were not scored. The total score was calculated as the sum of the number of cells scored for each class times that class value.

### Micronucleus test in peripheral blood

The micronucleus assay in peripheral blood was performed according to Hayashi *et al.* (1990) with modifications by Oliveira *et al.* (2009a). A 20 μL aliquot of peripheral blood was collected at 24, 48 and 72 h after treatments. Blood samples were placed on a slide previously covered with 20 μL of acridine orange (1.0 mg/mL). Then, a coverslip was placed over the biological material and the slide was stored in a freezer (-20 °C) for a minimum period of seven days. A total of 2,000 reticulocytes were examined per animal by epifluorescence microscopy (Bioval®, Model L 2000A) at 400x magnification and filter settings as described above.

### Splenic phagocytosis assay

A spleen fragment (approximately 1/3 of the organ size) was macerated in physiological saline (0.9% NaCl), and 100 μL of a cell suspension was placed on a slide previously treated with 20 μL of acridine orange (1.0 mg/mL) and covered with a coverslip. Slides were stored in freezer until analysis. Epifluorescence microscopy analyses of a total of 200 cells per animal were conducted as described above. The presence or absence of phagocytosis was determined based on the reports by Hayashi *et al.* (1990), with modifications (Ishii *et al.*, 2011).

### Apoptosis assay

The morphological analysis of apoptosis was performed using 100 μL of a solution of homogenized spleen, liver, or kidney preparation. The slides were fixed in *Carnoy* solution for 5 min and then subjected to successively decreasing concentrations of ethanol (95%, 75%, 55% and 25%). Finally, they were rinsed with McIlvaine buffer for 5 min, stained with acridine orange (0.01%) for 5 min and rinsed again with buffer. Apoptotic cells (among a total of 100 cells/animal) were identified through analysis of DNA fragmentation patterns according to Mauro *et al.* (2011) with modifications (Navarro *et al.*, 2014). Epifluorescence microscopy analyses were conducted as described above.

### Percent damage reduction (%DR) and statistical analysis

Percent damage reduction was calculated according to Manoharan and Baneriee (1985) and Waters *et al.* (1990) as:

$$\%\text{DR}=\frac{{\text{M}}_{\text{pc}}-{\text{M}}_{\text{cg}}}{{\text{M}}_{\text{pc}}={\text{M}}_{\text{nc}}}\times 100$$

where M_pc_ = Mean of positive control, M_cg_ = Mean of combination group and M_nc_ = Mean of negative control.

This parameter enables to infer the chemopreventive capacity of a substance when in combination with a known mutagenic substance. Values were expressed as the mean ± standard error of the mean (SEM), and the data were analyzed by analysis of variance (ANOVA) followed by a Tukey's post-hoc test using GraphPad Prism software (version 3.02; Graph-Pad Software Inc., San Diego, CA, USA). The significance level was set at p < 0.05.

## Results

### Isolation of cardanol

Extraction of saturated chain cardanol from cashew nut shell liquid was based on Srinivasa *et al.* (2002), however the required degree of purity was not reached. For this reason, liquid column chromatography was performed to assess the purity of the cardanol mixture, which resulted in a yield of 26% on a weight basis. Finally, a yield of 61% on a weight basis was reached by catalytic hydrogenation of cardanols. Cardanol, 3-pentadecylphenol was then purified by liquid column chromatography, and the purity of the compound for biological activity assays was reinforced by an additional recrystallization process in 90% ethanol, yielding a pure white solid (melting point 51-52 °C). Purity was confirmed by ^1^H-NMR spectroscopy at 300-MHz frequency in deuterated chloroform (CDCl_3_) (Supplementary Material 1). The spectroscopic data were compared to data already reported in the literature, confirming the purity of the product.

### Biometric parameters of animals exposed to cardanol

**Figure 3:**
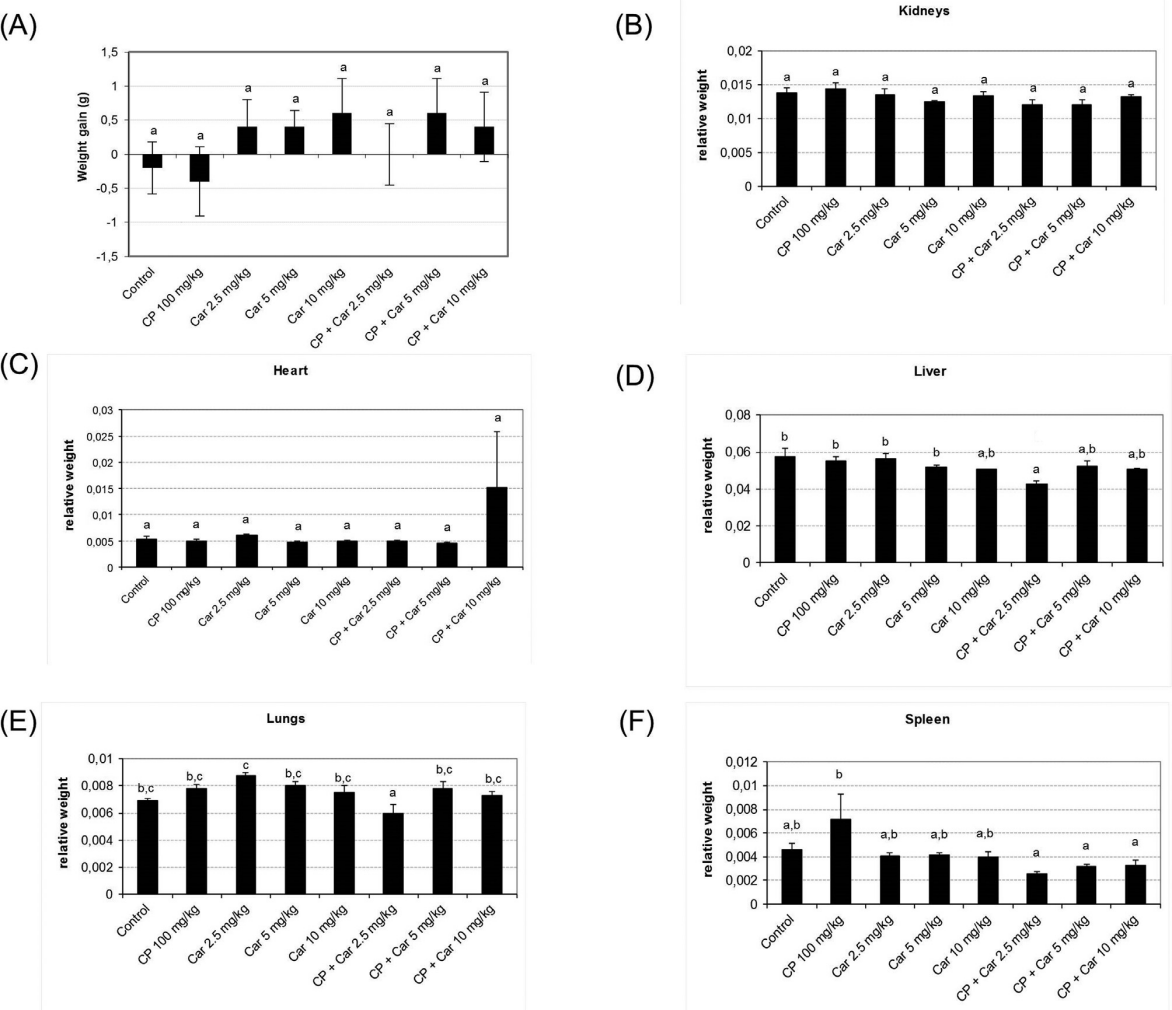
Weight gain and relative weights of organs from animals treated with cardanol alone or in combination with cyclophosphamide. (A) Weight gain of animals exposed to cardanol; weight gain was calculated by the difference between animal weigh after treatments and before treatments. Relative weights of kidneys (B), heart (C), liver (D), lungs (E) and spleen (F); relative weight was calculated as the ratio of each organ's absolute weight to the animal's weight. Bars represent the mean ± SEM. Different letters represent statistically significant differences (ANOVA followed by Tukey's post-hoc tests; p ≤ 0.05).

No significant differences were observed in the weight gain of the animals (Figure 3A), absolute and relative weight of the kidneys, heart and spleen, when compared with the control groups (Figure 3B,C,F). However, the group that was treated with cyclophosphamide combined with cardanol at the dose of 2.5 mg/kg showed a decrease in the relative weight of the liver (Figure 3D), when compared to the control, cyclophosphamide and cardanol 2.5 / 5 mg/kg groups, and in the relative weight of the lungs (Figure 3E) when compared to all groups.

### Comet and micronucleus assays

Data obtained from the comet assay showed that cardanol increased the frequency of damaged cells 2.02, 1.74 and 1.63 times, respectively, for the doses of 2.5, 5 and 10 mg/kg. Thus, we observed an inversed dose-response curve, and only the lowest dose was statistically significant. However, when observing the score of comet classes (Table 2), cardanol showed absence of genotoxicity. All cardanol doses where combined with cyclophosphamide, because there was absence of genotoxicity. Thus, in the combination groups, antigenotoxic activity was observed with percentages of damage reduction of 31.76, 18.90 and 18.90 for the doses of 2.5, 5 and 10 mg/kg, respectively (Table 2).

**Table 2:** Means ± SEM of damaged cells, distribution between damage classes, and scores related to antigenotoxicity tests of cardanol by means of the comet assay. Different letters indicate statistically significant differences (p ≤ 0.05; ANOVA and Tukey's post-hoc test).

Treatments	Mean frequency of cells with DNA damage	Classes				Score	% Damage reduction
		0	1	2	3		
Control	15.2 ± 2.26^a^	84.8 ± 2.26	9.8 ± 2.15	3.8 ± 0,66	1.6 ± 0.81	22.2 ± 3.2^a^	-
CP 100mg/kg	91.4 ± 1.86^d^	8.6 ± 1.86	26.4 ± 1.28	39.6 ± 3.66	25.4 ± 2.67	181.2 ± 4.7^d^	-
Car 2.5mg/kg	30.8 ± 1.43^b^	69.2 ± 1.43	23.8 ± 1.11	5.2 ± 0.58	1.8 ± 0.37	39.6 ± 2.6^a^	-
Car 5mg/kg	26.4 ± 2.0^a^	73.6 ± 2.04	17.4 ± 1.21	6.8 ± 2.71	2.2 ± 0.73	37.6 ± 5.7^a^	-
Car 10mg/kg	25.4 ± 1.72^a^	74.6 ± 1.72	20.6 ± 2.54	3.0 ± 0.83	1.8 ± 0.73	32.0 ± 1.4^a^	-
CP + Car 2.5mg/kg	67.2 ± 6.91^c^	32.8 ± 6.91	63.4 ± 7.06	1.4 ± 0.51	2.4 ± 0.40	73.4 ± 7.0^b^	31.76%
CP + Car 5mg/kg	23.0 ± 3.62^c^	23.0 ± 3.62	66.0 ± 3.70	3.6 ± 1.03	7.4 ± 1.03	95.4 ± 4.0^b^	18.90%
CP + Car 10mg/kg	23.0 ± 2.77^c^	23.0 ± 2.77	57.4 ± 2.65	7.8 ± 1.06	11.8 ± 1.59	108.4 ± 5.7^c^	18.90%

Control: Experimental group of untreated animals;

CP 100 mg/kg: Experimental group that received cyclophosphamide at the dose of 100mg/kg;

Car 2.5 mg/kg: Experimental group that received cardanol at the dose of 2.5 mg/kg;

Car 5 mg/kg: Experimental group that received cardanol at the dose of 5 mg/kg;

Car 10 mg/kg: Experimental group that received cardanol at the dose of 10 mg/kg;

CP + Car 2.5 mg/kg: Experimental group that received cyclophosphamide (100 mg/kg) and cardanol at the dose of 2.5 mg/kg;

CP + Car 5 mg/kg: Experimental group that received cyclophosphamide (100 mg/kg) and cardanol at the dose of 5 mg/kg;

CP + Car 10 mg/kg: Experimental group that received cyclophosphamide (100 mg/kg) and cardanol at the dose of 10 mg/kg.

**Figure 4:**
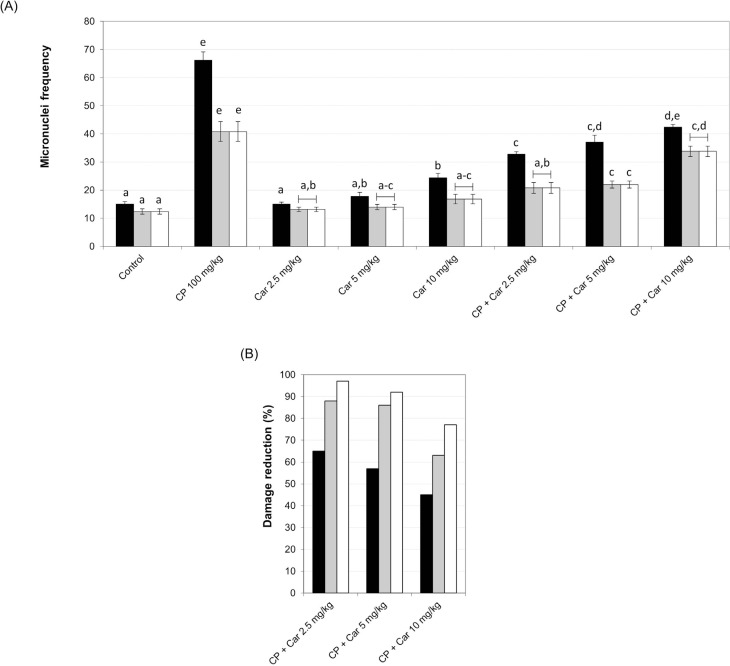
Evaluation of the mutagenic and antimutagenic potential of cardanol. (A) Means ± SEM of micronuclei frequency. (B) Percentage reduction of mutagenic damage. Bars represent different time points of analysis: Black bars 24 h, gray bars 48 h and white bars 72 h after the treatment. Different letters represent statistically significant differences (ANOVA followed by Tukey's post-hoc tests; p ≤ 0.05).

In turn, the micronucleus assay showed that only the 10 mg/kg cardanol dose increased micronuclei frequency after 24 h of treatment compared to the respective control, but not at 48 h and 72 h (Figure 4A). The results showed cardanol presented protective activity: it decreased micronucleus frequency, and damage reduction was greater at cardanol 2.5 mg/kg at all time points examined (Figure 4A). DNA damage reduction was significant at all doses tested and at all time points, and %DR values were 65, 57 and 45% after 24 h; 88, 86 and 63% after 48 h; and 97, 92 and 77% after 72 h of treatment for doses of 2.5, 5 and 10 mg/kg, respectively (Figure 4B).

### Splenic phagocytosis and apoptosis assay

**Figure 5:**
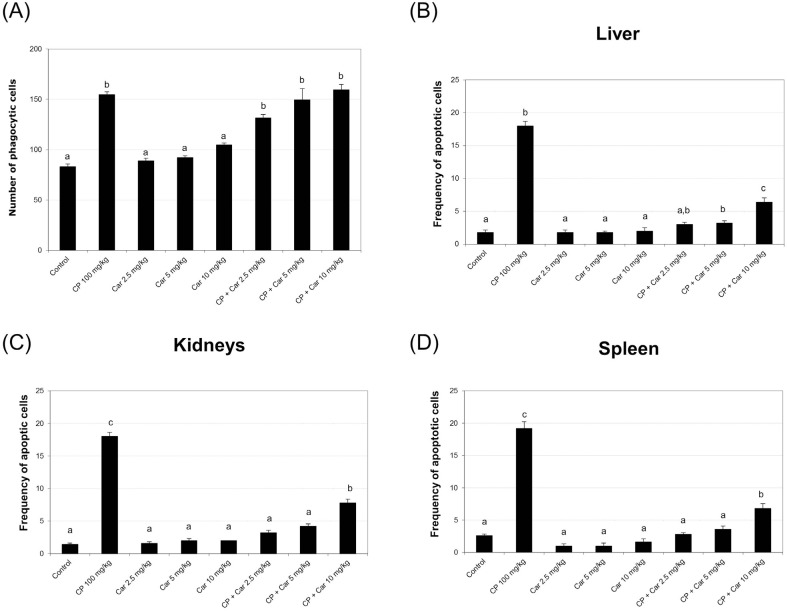
Phagocytic and apoptotic cells. Number of phagocytic cells (A). Number of apoptotic cells in the liver (B), kidneys (C), and spleen (D). Bars represent the mean ± SEM. Different letters represent statistically significant differences (ANOVA followed by Tukey's post-hoc tests; p ≤ 0.05).

Cardanol induced no change in phagocytosis rates when assessing splenic phagocytosis in animals treated with cardanol. In turn, treatments with cyclophosphamide isolated or in combination with cardanol, showed increased phagocytosis (Figure 5A).

When assessing whether cardanol induced apoptotic cell death, the results showed that cardanol alone did not increase apoptosis in liver, kidneys or spleen. However, a reduction in the number of apoptotic cells was observed in all organs studied in the treatments combined with cyclophosphamide (Figures 5B-D).

## Discussion

Cardanol has been previously described as an important antioxidant and antimutagenic compound (Melo Cavalcante *et al.*, 2003; Rodrigues *et al.*, 2006; Trevisan *et al.*, 2006; De Lima *et al.*, 2008). Cardanol and cardol also induced cytotoxicity and cell death without DNA fragmentation in cancer cells, which suggests that these compounds could be alternative antiproliferative agents (Teerasripreecha *et al.*, 2012).

The score of the comet assay showed that cardanol is not genotoxic. However, this compound can increase the frequency of cells with DNA damage in an inverse dose-response curve. In other words, the lower the dose, the greater the occurrence of genotoxic damage. When analyzing the micronucleus assay data at 24h, there was a directly proportional relation between the increase in the dose and mutagenicity. Thus, in this case, there was a dose-response correlation, and only the higher dose demonstrated toxicity. At 48 and 72h, the same pattern of dose-response was observed, but all doses showed absence of mutagenicity. Considering these results, it is inferred that cardanol, according to our experimental design, has low capacity to induce DNA damage, and this fact stimulated the continuation of the study. Cardanol is known to have antioxidant and antimutagenic proprierties (Melo Cavalcante *et al.*, 2003; Rodrigues *et al.*, 2006; Trevisan *et al.*, 2006; De Lima *et al.*, 2008) and it is cytotoxic for tumor cells (Teerasripreecha *et al.*, 2012; Patel, 2016). Thus we also evaluated its chemopreventive and/or its ability to potentiate damage caused by chemotherapy. All doses showed to have antigenotoxic and antimutagenic potential, and an inverse correlation dose-response was observed. This data showed the chemopreventive capacity of cardanol. An interesting fact is that when a dose is at the limit of genotoxicity, chemopreventive activity is also observed. This was not expected, however, it is common to find similar results, as reported by Oliveira *et al.* (2013), who examined β-glucan activity, noting that this agent may be both genotoxic and antigenotoxic. In addition, similar data were reported for shiitake (*Lentinula edodes* (Berkeley) Pegler; Miyaji *et al.*, 2004) and *Caesaria sylvestris* extracts (Oliveira *et al.*, 2009b).

The micronucleus assay showed that cardanol at 2.5 mg/kg did not increase the number of cells with DNA damage at the time points studied, and it provided the best protection against DNA damage, as observed by %DR. This result indicated that genotoxic lesions observed in the comet assay were not fixed in the DNA as a permanent DNA damage. Rather, lesions detected by this comet assay, including single- and double-strand breaks, alkaline-labile sites, crosslinks, excision repair sites, methylation damage and adducts (Singh, 2000; Tice *et al.*, 2000; Hermeto *et al.*, 2014), may be repaired without becoming mutations (Oliveira *et al.*, 2007). In contrast, the micronucleus assay evaluates aneugenic and clastogenic activities that are not prone to repair. Micronuclei originate from acentric chromosome fragments, acentric chromatid fragments, or whole chromosomes that fail to be included in the daughter nuclei at the completion of telophase during mitosis. These chromosomes or fragments are enclosed by a nuclear membrane and present a morphology similar to nuclei, except for their smaller size (Fenech *et al.*, 2011).

Studies have reported that there are two mechanisms that may explain antimutagenesis: bioantimutagenesis and desmutagenesis. In bioantimutagenesis, an antioxidant is able to modulate DNA repair and replication through enzymes. In turn, in desmutagenesis, a compound adsorbs another, thus preventing its action (Kada *et al.*, 1982; Kada and Shimoi, 1987; De Flora, 1998; Duarte *et al.*, 2009; Oliveira *et al.*, 2006, 2007, 2009a, 2013, 2014). Antimutagenesis through mechanisms of desmutagenesis and bioantimutagenesis could explain our damage reduction data, since treatments were administered simultaneously, *i.e.*, the damage-inducing agent and cardanol were administered sequentially.

Cardanol is not able to induce splenic phagocytosis, and its combination with cyclophosphamide caused no change in the levels found in the corresponding control (positive control - cyclophosphamide). These results suggest that cells with cardanol-induced damage experienced no phagocytosis. Thus, the compound did not demonstrate immunomodulatory activity.

When assessing cardanol-induced cell death, the results showed cardanol alone failed to induce apoptosis in liver, kidneys and spleen, although apoptosis reduction occurred in treatments combined with cyclophosphamide. This reduction in cell death occurred more efficiently at cardanol 2.5 mg/kg combined with cyclophosphamide. In general, apoptotic cell death is presumably triggered when cells experience extensive damage that cannot be repaired. Clastogenic and aneugenic damage, evidenced in micronuclei, could also be eliminated by apoptosis (Fenech *et al.*, 1999). However, our results showed that cardanol increased micronuclei frequency, albeit not that of apoptosis. According to Vukicevic *et al.* (2004), this may result from possible apoptotic suppression in micronucleated cells, which become necrotic even before undergoing mitosis.

According to Damasceno *et al.* (2002), Freitas *et al.* (2005), Brugiolo *et al.* (2010) and Gonçalves *et al.* (2013, 2014), the weight gain and relative weight of the organs during the experimental period can be used as toxicity parameters. Biometric parameters from the present study support the fact that cardanol lacks toxicity, when administered alone or in combination with cyclophosphamide. There was no statistically significant variation in weight gain and relative weight of the kidneys, heart and spleen. However, statistically significant differences were observed in the liver and lungs for the group that received cyclophosphamide combined with the lowest dose of cardanol. A possible explanation to this fact is that in this specific group there was no weight gain during the experimental period. Furthermore, this group had the highest standard error, indicating higher rate variation in the size of the animals, even after random distribution. Thus, we consider that this data is not biologically relevant.

A final interpretation of the data showed that, despite its chemoprotective capacity, cardanol has a tendency to induce DNA damage, and hence, caution is needed if it is used as a chemopreventive agent. Moreover, when combined with cyclophosphamide, this compound reduced the frequency of apoptotic cells. Thus, this compound is likely not to be used as an adjuvant in chemotherapy treatments that use cyclophosphamide.

## Supplementary material

The following online material is available for this article:

Figure S1 - ^1^H-NMR spectrum of cardanol (CDCl_3_, 300 MHz)

This material is available as part of the online article from http://www.scielo.br/gmb.
