# Supplementary figures and images for: Cardanol: toxicogenetic assessment and its effects when combined with cyclophosphamide
Source: Genet Mol Biol. 2016 Apr-Jun;39(2):279–89. doi: 10.1590/1678-4685-GMB-2015-0170 (PMC4910563; doi:10.1590/1678-4685-GMB-2015-0170)

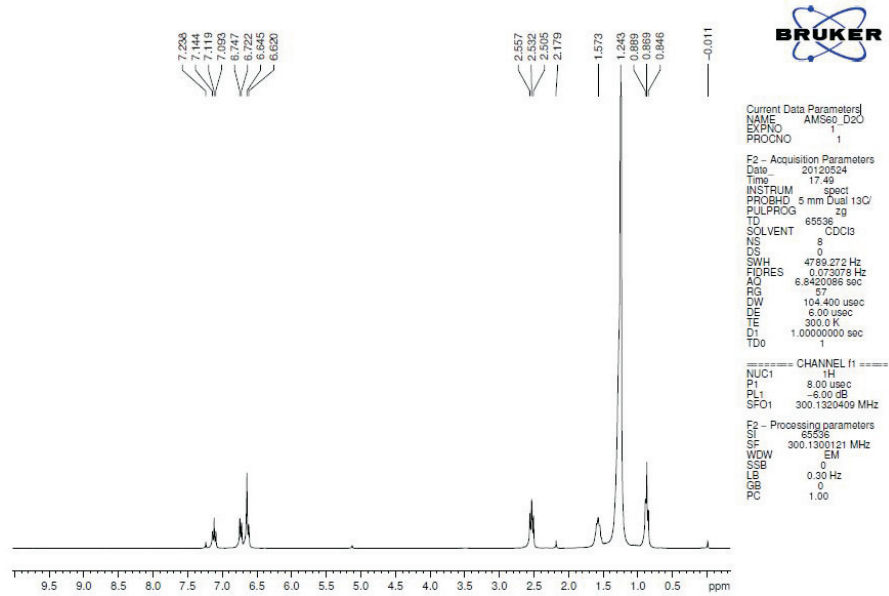

**Figure S1** -  $^1\text{H}$ -NMR spectrum of cardanol ( $\text{CDCl}_3$ , 300 MHz)

Supplement: Supplementary file 1 [file 1415-4757-gmb-39-02-0279-Suppl01.pdf]
